# Single-Cell High-Throughput Technologies in Cerebrospinal Fluid Research and Diagnostics

**DOI:** 10.3389/fimmu.2019.01302

**Published:** 2019-06-11

**Authors:** Tobias V. Lanz, Anne-Katrin Pröbstel, Iris Mildenberger, Michael Platten, Lucas Schirmer

**Affiliations:** ^1^Department of Neurology, Medical Faculty Mannheim, University of Heidelberg, Mannheim, Germany; ^2^Division of Immunology and Rheumatology, Department of Medicine, Stanford University School of Medicine, Stanford, CA, United States; ^3^Department of Neurology, Weill Institute for Neurosciences, University of California, San Francisco, San Francisco, CA, United States; ^4^Departments of Medicine and Biomedicine, Neurologic Clinic and Policlinic, University Hospital Basel, University of Basel, Basel, Switzerland; ^5^DKTK Clinical Cooperation Unit Neuroimmunology and Brain Tumor Immunology, German Cancer Research Center (DKFZ), Heidelberg, Germany

**Keywords:** cerebrospinal fluid (CSF), RNA sequencing (RNAseq), repertoire sequencing, single cell gene expression, mass spectrometry, flow cytometry

## Abstract

High-throughput single-cell technologies have recently emerged as essential tools in biomedical research with great potential for clinical pathology when studying liquid and solid biopsies. We provide an update on current single-cell methods in cerebrospinal fluid research and diagnostics, focusing on high-throughput cell-type specific proteomic and genomic technologies. Proteomic methods comprising flow cytometry and mass cytometry as well as genomic approaches including immune cell repertoire and single-cell transcriptomic studies are critically reviewed and future directions discussed.

## Introduction

**Figure 1:**
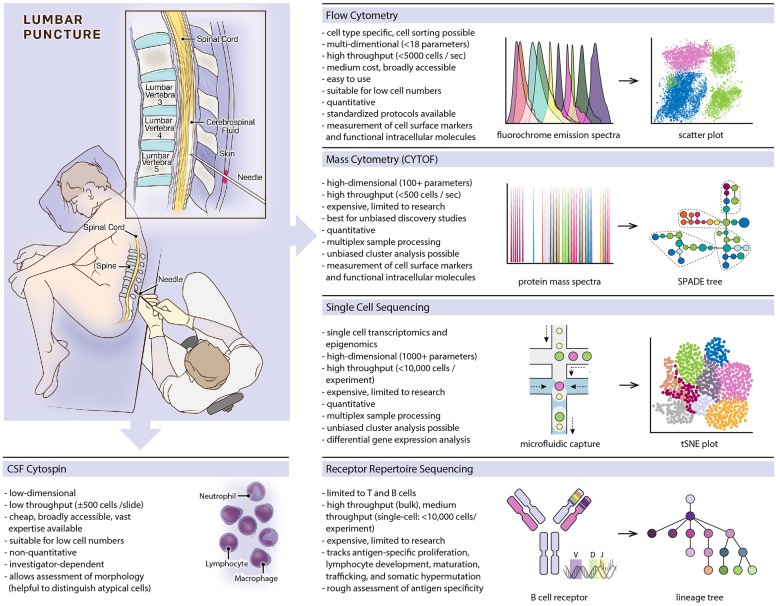
Overview of featured single-cell methods, listing prominent characteristics of each method and providing schematic depictions of methodological workflows and typical data visualizations.

Since its inception by Heinrich Quincke (1), lumbar punctures and cerebrospinal fluid (CSF) analyses have become invaluable diagnostic tools in the clinical care of neurological patients. Early-on, microscopic examination of CSF cells was included in the work-up and facilitated the diagnosis of inflammatory and tumorous diseases of the central nervous system (CNS). Quincke subclassified CSF cells into leukocytes, red blood cells and epithelial cells (2). Routine work-ups include cell counts and detailed microscopic examinations with cells spun onto glass slides and characterized by May–Gruenwald–Giemsa stain allowing differentiation of red blood cells, lymphocytes, monocytes, granulocytes, and detection of malignant cells (Figure 1). Red blood cells and leukocytes can be further assessed for activated cellular states (plasmablasts, activated macrophages), and associated with certain diseases (erythrophages, siderophages, lipophages) (3). Introduction of labeled antibodies against cell-specific antigens in the 1960s allowed detailed analysis on slide-bound CSF cells by immunofluorescence and enzyme-linked immunocytochemistry (4–6). However, traditional microscopic assessment exhibits several limitations: (i) microscopic examinations are supervised, investigator-biased, and must be carried out by experienced personnel; (ii) throughput is low as specimens are spun separately on single slides; (iii) sensitivity is low, in particular for rare cell populations; (iv) quantitative analyses are challenging (Figure 1).

Here, we focus on next-generation high-throughput technologies allowing cell-type specific analyses with high accuracy in a fast and quantitative manner. Currently, most methods are used in research requiring expensive equipment and experience in sample preparation and computational data analysis. Focused efforts are necessary to translate findings from exploratory research into clinical practice, making those high-throughput methods broadly accessible. Obstacles include low cell counts and a short life span of CSF cells, posing challenges for biobanking. Study inclusion, sample collection, quality check (e.g., blood cell contamination, RNA integrity), and sample processing must be done quickly according to standardized protocols. Hence, we advocate for including novel single-cell technologies in future studies enabling their use on a broader scale and thereby increasing the relevance of CSF cytology in clinical settings.

### Profiling CSF Cell Heterogeneity by High-Throughput Flow Cytometry

Multicolor flow cytometry was developed in the 1960s (7), became available for broader use in the 1970s, and revolutionized immunological research, biomarker development, and clinical diagnostics (8, 9) (Figure 1). Fluorescently labeled cells get excited by multiple lasers, and the detection of fluorochrome emissions allows a multi-parameter cell-type specific characterization. Modern cytometers can detect up to 18 fluorochromes in parallel and process several thousand cells per second. In addition to cell surface markers, intracellular molecules can be detected, revealing functional cellular states like influx of ions, expression levels of transcription factors, phosphorylation states, and cytokine levels (10, 11). Fluorescence-activated cell sorting partitions cell populations for downstream analyses including RNA sequencing and cell culture assays.

Flow cytometry has been implemented for detailed cell analyses including T cell counts in HIV, immunophenotyping in immunodeficiencies, hematological malignancies, and during stem cell transplantation (12, 13). Flow cytometric CSF analysis still lags behind due to high variabilities in cell populations, lack of disease-specific cell markers, and low cell counts in many neurological diseases. False-negative results are common in disorders with only subtle increase in cell numbers, but higher sensitivities can be achieved by increasing CSF volumes and repeated measurements (14, 15). CSF cell populations in healthy individuals are relatively uniform, however, differ significantly from cell distributions in blood (16–18). T cells are the most abundant cell type in the CSF, the CD4/CD8 ratio is skewed toward CD4^+^ (CSF: 3 vs. blood: 2.1), and CCR7^+^ central memory T helper subtypes are the dominating phenotype (~90% of the CD3+CD4+ T cell population), suggesting an important role in immune surveillance of the CNS under healthy conditions (17); granulocyte, B and NK cell counts are low (<1%) (19).

Most validated disease-specific flow cytometry panels are currently used in primary CNS lymphomas supplementing microscopic cytology and adding a high positive (92%), however, low negative predictive value (52%) (15, 20). So far, studies have not determined predictive values of CSF flow cytometry in non-malignant diseases preventing its use in routine diagnostics of neuroinflammatory, neurodegenerative, and neurovascular disorders. However, many exploratory studies have described disease-specific features, and more advanced granular flow panels will help establish flow cytometry as a valid diagnostic tool.

Elevated CD4/CD8 ratios have been described in stroke, Guillain-Barré syndrome and multiple sclerosis (MS) and low CD4/CD8 ratios in HIV. B cells and activated plasmablasts are elevated in infectious conditions including HIV and Lyme disease as well-autoimmune diseases like MS (21, 22), while monocyte counts are low in these diseases but elevated in glioblastoma patients (23, 24). Notably, NK cells have been reported to be elevated in patients with viral meningitis (24). Several studies on inflammatory diseases have used flow cytometry for more precise phenotypical profiling of T cell subsets, such as CD8^+^ cytotoxic or γδ-TCR-positive T cells (25–29), and NK cells (17, 19, 30), and some studies could correlate findings to treatment responses or disease progression (31, 32). CSF cells in primary neurodegenerative diseases are less well-studied (vs. proteins such as neurofilaments, tau, and amyloid that are enriched in the CSF) with some studies suggesting leukocyte activation in neurodegenerative disorders. For example, CD8^+^HLA-DR^+^ activated T cells correlate with neurocognitive decline in patients with Alzheimer's disease (33). Despite ample evidence that an active immune response contributes to neuronal damage after ischemic stroke, CSF flow cytometry seems to be of limited value in CNS ischemia. A larger flow cytometry study recently reported a slight increase in cell numbers without differences in cell distributions when compared to healthy individuals, irrespective of stroke size and location (34). In summary, exploratory studies have defined flow cytometry panels for several neurological diseases. CSF flow cytometry is particularly valuable in oncological diseases, followed by inflammatory and infectious disorders. Correlations between cell status and clinical outcome can provide meaningful support for neurological diagnosis and patient care. Defining additional granular marker panels will likely increase its relevance and justify a broader use in CSF diagnostics in the future.

### Characterizing CSF Cell Subsets by High-Dimensional Mass Cytometry

Mass cytometry (cytometry by time of flight, CYTOF) is related to flow cytometry but uses metal ion labels instead of fluorochromes. Individual cells vaporize in inductively coupled argon plasma with metal ions getting ionized and introduced into a time-of-flight (TOF) mass spectrometer allowing to distinguish isotopes by a single atomic mass units (35). With virtually no overlap between mass spectra, multidimensional data acquisition of more than 100 parameters per cell is possible (usually in the range of 30 to 60) allowing a throughput of up to 500 cells per second (Figure 1). Data deconvolution algorithms provide solutions for dimensionality reduction and clustering. Common methods include principal component analysis (PCA) (36), t-distributed stochastic neighbor embedding (t-SNE) (37), uniform manifold approximation and projection (UMAP) (38), spanning-tree progression analysis of density-normalized events (SPADE) (36) and cluster identification, characterization, and regression (CITRUS) (39). With the expansion of simultaneously detected parameters, cell characterization is possible at an unprecedented granular level, and intracellular molecular labeling further enables dynamic monitoring of functional markers that add mechanistic insight to descriptive cellular states (40, 41), and even simultaneous measurement of specific RNA and protein expression levels in single cells being possible (42).

CYTOF has been utilized to map the cellular landscape of neuronal, glial and immune cells in rodent brains. For example, CD44 was identified as a potential marker for infiltrating leukocytes, border-associated macrophages could be distinguished from microglia and dendritic cells, and a new CD317^+^MHCII^+^CD39^+^CD86^+^ microglia subset was identified in neurodegenerative and inflammatory models (41, 43, 44). Protocols to dissociate and measure tumor cells and tumor infiltrating leukocytes from glioma have been tested, and larger CYTOF studies from human brain tumor tissue can be expected soon (45). Recently, peripheral blood mononuclear cell (PBMC) populations of glioblastoma and narcolepsy patients have been studied by CYTOF (40, 46) and highlighted the role of immune cells. However, due to low cell counts and freeze-storing challenges, CYTOF studies have not yet been performed on CSF. CYTOF is more expensive and challenging than flow cytometry with computational expertise necessary to evaluate high dimensional data. Currently, it is still an research tool, but the myriad of investigated parameters can be condensed to a focused set of cellular markers to be adopted for flow cytometry and used to design cell-specific therapies.

### Understanding CSF Lymphocyte Diversity by Immune Repertoire Sequencing

B and T cell receptors (BCR and TCR) exhibit unique genetic characteristics that can serve as natural markers of the adaptive immune system. BCR and TCR are specialized cell surface receptors on B and T lymphocytes, respectively, determining adaptive immune responses and immune memory (Figure 1). Soluble BCRs are secreted as immunoglobulins, which opsonize free antigens and activate complement factors as well as innate immune cells. Cross-ligation of the membrane-bound BCR by antigens triggers B cell activation and proliferation. T cells detect specific antigens via TCRs when presented on major histocompatibility complexes (MHC) by antigen-presenting cells. During lymphocyte development, the genes coding for each lymphocyte's BCR and TCR rearrange and mutate, resulting in an astounding diversity of 10^13^-10^18^ possible BCRs and TCRs (47–49), although the realized lymphocyte repertoire of an individual is several magnitudes smaller (50). High diversity is needed to defend against a vast number of possible pathogens. B cells (but not T cells) continue to mutate their BCR upon B cell activation, striving to further increase affinity to its cognate antigen in a process called somatic hypermutation. BCR and TCR gene signatures are unique to each lymphocyte and passed on to descendant cells. The entirety of a person's BCR and TCR sequences comprises the immune repertoire, which can be studied using DNA or mRNA next-generation sequencing methods (51–53). Repertoire analysis is challenging because high sequence variabilities complicate alignments to germline sequences. It therefore requires rigorous validation to differentiate mutations from sequencing errors. As each lymphocyte carries one unique receptor sequence, single-cell conclusions can be drawn even from bulk-sequencing experiments. However, single-cell sequencing is needed to describe a receptor in its entirety, as each receptor consists of two hetero-dimerizing protein chains (53). Direct inference of an antigen from the receptor sequence is currently not possible, however, new methods allow clustering TCRs with similar antigen-specificities based on predicted structures of antigen binding sites (54). Repertoires provide valuable information about lymphocyte development and maturation, somatic hypermutation, lymphocyte trafficking (55, 56), and malignant transformations (57). Hence, several studies have suggested the use of repertoires as disease-specific biomarkers in MS, CNS lymphomas, and other neurologic diseases (58–60).

B cell repertoire sequencing has recently attracted major attention in MS when clinical trials using B cell depleting therapies showed enormous efficacy (61, 62). Studies comparing CSF, blood, lymph nodes, and meningeal B cell follicles have suggested that B cells mature in secondary lymphoid organs and traffic across the blood brain barrier as switched memory B cells and plasmablasts (55, 56, 63). Other repertoire studies demonstrated overrepresentation of heavy chain V gene family 4 (VH4) in the CSF of MS patients, likely as a result of chronic antigen-specific B cell activation and proliferation. Specific VH4 genes together with a set of characteristic mutations were proposed as an experimental biomarker for MS (58, 60, 64, 65). Besides MS (66–69), BCR and TCR repertoire sequencing of CSF lymphocytes have been performed to tackle similar questions in other neuroimmune diseases including NMDA and LGI1-antibody positive encephalitis (70, 71), Rasmussen encephalitis (72) and glioma (73). However, larger studies are needed to recommend CSF repertoire sequencing to be used in clinical neuroimmunology.

### Dissecting CSF Cellular and Molecular Heterogeneity by Single-Cell Genomics

Single-cell sequencing has emerged rapidly over the last years and provides multi-dimensional and high-throughput possibilities to study cell-type specific diversity based on cellular transcriptomes (Figure 1). Plate-bound (several 100 cells per experiment), droplet-bound, and multifluidic-based (several thousand cells per experiment) methods provide sequencing depths of ~1,000 to ~6,500 genes per cell (74, 75). Single-cell RNA-sequencing (scRNA-seq) can be performed using both fresh cell suspensions from liquid and solid tissue samples (76–79) as well as isolated nuclei from frozen material with well-preserved RNA (80–82). scRNA-seq allows studying the entire transcriptome in an unbiased manner, dissecting both cellular diversity and molecular transcriptomic changes in individual cells. This becomes an extremely powerful tool when identifying disease-related cell populations or performing repeated sampling during the course of a disease.

scRNA-seq had great influence on immunological research by enabling the identification of specific immune cell subtypes and fostering our understanding of cellular diversity and cell-type specific regulation patterns (78, 79, 83–85). Recently, elegant computational algorithms have successfully inferred BCR and TCR repertoires from scRNA-seq data (86–88). While single-cell genomic methods have been successfully applied to solid tissues using animal models and human pathologies including glioma and MS (82, 89–91), it yet has only been the subject of very few scRNA-studies focusing on HIV (92) and MS (93) suggesting the presence of disease-specific myeloid (HIV) and T follicular helper cell (MS) subtypes in the CSF.

A broader availability and a wider use of scRNA-seq have so far been impeded by high costs of reagents and the need for computational expertise to run standardized high-performance analyses. Also, due to the relatively low cell number in non-infectious CSF preparations, high-throughput scRNA-seq technologies have been restricted to solid tissue or liquid biopsies like blood, where high cell numbers are available. Novel multiplex approaches, however, can significantly reduce costs and overcome challenges related to low CSF cell number input by barcoding and pooling cells from different individuals to be distinguished in retrospect during data analysis. In an effort to improve multiplex approaches, natural genomic variations, such as single-nucleotide polymorphisms (SNPs), can be exploited to exclude droplets containing more than one cell based on their inter-individual genomic signature (94). Another multiplex assay uses lipid-tagged indices to identify cells from different individuals and applies this method to single-cell preparations (95).

In addition, single-cell epigenetic technologies were recently developed that sequence the open chromatin landscape of individual cells. These methods, which include ChIP-seq (chromatin immunoprecipitation DNA-sequencing) and ATAC-seq (assay for transposase-accessible chromatin using sequencing) (96, 97), have become powerful tools to profile immune and tumor cell subsets in health and disease, in particular when used with other methods like single-cell repertoire sequencing (98, 99). Combinations of single-cell genomic methods in conjunction with high-throughput multiplex strategies will change biomedical research dramatically in the near future and help dissect cellular heterogeneity and cell-type specific gene regulation and expression in an unprecedented way (100).

## Conclusion and Future Directions

We introduce four high-throughput multi-parameter technologies and advocate for their implementation in CSF cell diagnostics to gain a deeper understanding of cellular, proteomic, and transcriptomic changes on a single-cell level. The unparalleled depth of these methods allows researchers to describe precise cellular landscapes of organ systems in health and disease, characterize specific cell subsets in vast detail, perform network analyses in complex cellular systems, and suggest new cellular biomarkers for pathologies (41, 85, 101). Currently, only flow cytometry has been introduced in routine clinical CSF diagnostics. However, its relevance is often limited and larger datasets with standardized protocols are needed to maximize its contribution to CSF diagnostics. Mass cytometry, repertoire sequencing, and single-cell transcriptomics/epigenomics are still experimental methods, ideally suited to gain detailed unbiased overviews and to provide critical insight into disease mechanisms. Large high-dimensional datasets derived from these methods need to be condensed to focused marker sets that can be measured routinely. Notably, additional single-cell technologies have been explored including genomic sequencing (102), single-cell metabolomics (103), and single-cell proteomics (104). Single-cell methods should be implemented in future clinical trials as they can add valuable mechanistic insight, and neurologists will have to monitor the maturation of these technologies in the near future as they promise to revolutionize cellular CSF diagnostics.

## Author Contributions

TL, A-KP, and LS conceptualized, wrote, and revised the manuscript. IM and MP contributed to writing the manuscript.

### Conflict of Interest Statement

The authors declare that the research was conducted in the absence of any commercial or financial relationships that could be construed as a potential conflict of interest.
